# BFSP1 C-terminal domains released by post-translational processing events can alter significantly the calcium regulation of AQP0 water permeability

**DOI:** 10.1016/j.exer.2019.02.001

**Published:** 2019-02-18

**Authors:** Antal Tapodi, Daniel M. Clemens, Alice Uwineza, Miguel Jarrin, Martin W. Goldberg, Emmanuelle Thinon, William P. Heal, Edward W. Tate, Karinne Nemeth-Cahalan, Irene Vorontsova, James E. Hall, Roy A. Quinlan

**Affiliations:** a Department of Biosciences, The University of Durham, South Road, Durham, DH1 3LE, UK; b Physiology and Biophysics, UC Irvine, Irvine, CA, USA; c Department of Chemistry, Molecular Sciences Research Hub, Imperial College London, Wood Lane, London, W12 0BZ, UK; d Institute of Chemical Biology, Molecular Sciences Research Hub, Imperial College London, Wood Lane, London, W12 0BZ, UK; e Biophysical Sciences Institute, The University of Durham, South Road, Durham, DH1 3LE, UK; 1 Department of Biochemistry and Medical Chemistry, University of Pecs 12, Szigeti H-7624, Pecs, Hungary.; 2 Verseon Corporation, 47071 Bayside Parkway, Fremont, CA 94538.; 3 Molecular Cell Biology of Autophagy Laboratory, The Francis Crick Institute, 1 Midland Road, London NW1 1AT, UK.; 4 King’s College London, 5–11 Lavington Street, London SE1 0NZ, UK.; 5 Contributed equally.

## Abstract

BFSP1 (beaded filament structural protein 1, filensin) is a cytoskeletal protein expressed in the eye lens. It binds AQP0 *in vitro* and its C-terminal sequences have been suggested to regulate the water channel activity of AQP0. A myristoylated fragment from the C-terminus of BFSP1 was found in AQP0 enriched fractions. Here we identify BFSP1 as a substrate for caspase-mediated cleavage at several C-terminal sites including D433. Cleavage at D433 exposes a cryptic myristoylation sequence (434–440). We confirm that this sequence is an excellent substrate for both NMT1 and 2 (N-myristoyl transferase). Thus caspase cleavage may promote formation of myristoylated fragments derived from the BFSP1 C-terminus (G434-S665). Myristoylation at G434 is not required for membrane association. Biochemical fractionation and immunogold labeling confirmed that C-terminal BFSP1 fragments containing the myristoylation sequence colocalized with AQP0 in the same plasma membrane compartments of lens fibre cells. To determine the functional significance of the association of BFSP1 G434-S665 sequences with AQP0, we measured AQP0 water permeability in *Xenopus* oocytes co-transfected with transcripts expressing both AQP0 and various C-terminal domain fragments of BFSP1 generated by caspase cleavage. We found that different fragments dramatically alter the response of AQP0 to different concentrations of Ca^2+^. The complete C-terminal fragment (G434-S665) eliminates calcium regulation altogether. Shorter fragments can enhance regulation by elevated calcium or reverse the response, indicative of the regulatory potential of BFSP1 with respect to AQP0. In particular, elimination of the myristoylation site by the mutation G434A reverses the order of water permeability sensitivity to different Ca^2+^ concentrations.

## 1. Introduction

Beaded filaments modulate the optical properties of the lens (Sandilands et al., 2003; Song et al., 2009), adjust its mechanical properties (Fudge et al., 2011) and influence the geometry of lens fiber cells (Gokhin et al., 2012; Maddala et al., 2011; Sandilands et al., 2003). Beaded filament structural proteins (BFSP1 and BFSP2) co-assemble to form filaments that closely associate with the protein chaperone, α-crystallin, (Carter et al., 1995). These filaments are enriched in the plasma-membrane-cytoskeleton complex and are often tightly apposed to the plasma membrane (FitzGerald, 1988; Sandilands et al., 1995a). The precise mechanism of this association with the plasma membranes is not well understood, but both BFSP1 and BFSP2 can bind the C-terminal domain of AQP0 (Lindsey Rose et al., 2006), an integral membrane protein, and both colocalize with AQP0 (Lindsey Rose et al., 2006). It was recently proposed that AQP0 could help anchor the EPPD complex (ezrin, periplakin, periaxin, and desmoyokin), a major site of integration for the lenticular cytoskeleton at the plasma membrane, through its interaction with ezrin (Wang and Schey, 2011).

Additionally some fragments of BFSP1 resist alkali extraction (Brunkener and Georgatos, 1992), a biochemical property associated with integral membrane proteins (Milks et al., 1988). In addition to *in vitro* evidence for the binding of BFSP1 to AQP0 (Lindsey Rose et al., 2006), EDC cross-linking studies of bovine lens membrane fractions found a cross-link between the D246 in AQP0 and K455 in BFSP1 (Wang and Schey, 2017), indicating that the C-terminal domain of BFSP1 participates in an interaction with AQP0. This interaction of BFSP1 with AQP0 could also be functionally significant. An arbitrarily selected C-terminal portion of BFSP1, not expected to arise from normal post-translational processing modestly increases the water permeability (P_f_) of AQP0 (Nakazawa et al., 2011). The ability of calcium to modulate the P_f_ of AQP0 in the presence of BFSP1 and its fragments was, however, not investigated. The interaction of AQP0 with other membrane associated lens proteins, and particularly BFSP1, is likely critical to understanding AQP0 function in the lens (Sindhu Kumari et al., 2015).

The principal post-translational modification of BFSP1 is proteolysis during both differentiation and ageing (Su et al., 2011). Peptide-specific antibodies show that fragments derived from the N- and C-terminal regions of BFSP1 localize to different sub-cellular compartments in lens fiber cells (Sandilands et al., 1995b). An internal cleavage was proposed to release the two regions (Sandilands et al., 1995b). The N-terminal region co-assembles with BFSP2 *in vitro* and forms intermediate filaments (Carter et al., 1995). The C-terminal region localizes to the plasma membrane (Sandilands et al., 1995b). Details of the enzymatic activities and the precise site of the proposed cleavage of BFSP1 remained elusive until the proteomic analysis of the urea-soluble protein fraction from lens membranes identified a myristoylated fragment derived from the C-terminal region of BFSP1 (Wang et al., 2010b). They noticed that a consensus myristoylation site in the sequence DVPDGGQISK immediately followed a caspase consensus site (Backes et al., 2005; Piippo et al., 2010). Post-translational myristoylation is often associated with the exposure of a cryptic site by caspase cleavage (reviewed in (Martin et al., 2011)). Caspase activity is not always linked to apoptosis, but is required during development and normal cell differentiation (Busso et al., 2010; Rudrapatna et al., 2013). Moreover such caspase activity can be readily detected in differentiating lens cells (Weber and Menko, 2005). Therefore whilst the mechanism for the revelation of this cryptic myristoylation motif and its role as a substrate for the N-methyltransferases expressed in the lens (Shrivastav et al., 2004) remain to be formally demonstrated (Wang et al., 2010b), these proteomic data have identified BFSP1 polypeptides derived from the C-terminal domain of BFSP1 that may well be regulators of AQP0.

In the lens, calcium and its regulation are critical to lens transparency (Maddala et al., 2013; Truscott et al., 1990). Both AQP0 (Ma et al., 2005) and BFSP1 (Marcantonio, 1992) are substrates for the calcium dependent protease, calpain. BFSP1 becomes a substrate when calcium is added to lens extracts or when lenses in culture were treated with a calcium ionophore to induce opacification by changing the calcium regulation (Marcantonio, 1992). Calcium regulates the water permeability of AQP0 *in vitro* (Reichow et al., 2013) (Nemeth-Cahalan and Hall, 2000; Nemeth-Cahalan et al., 2004) and probably also in the lens (Nemeth-Cahalan et al., 2013) through calmodulin (Reichow et al., 2013; Reichow and Gonen, 2008). Calcium also regulates the binding of A-Kinase Anchor Proteins (AKAP) binding to plasma membranes (Tao et al., 2006) and AQP0 activity is regulated by PKA phosphorylation (Gold et al., 2012; Németh-Cahalan et al., 2004). PKA mediated phosphorylation of AQP0 (now known to be facilitated by AKAP2) alters the manner in which calcium increases AQP0’s water permeability when expressed in *Xenopus* oocytes (Kalman et al., 2008). If serine 235 is phosphorylated, increasing the external calcium concentration from1.8 mM to 5 mM increases the water permeability. However if serine 235 is not phosphorylated, lowering the calcium concentration to 0 mM increases the water permeability (Gold et al., 2012). AQP0 phosphor-ylation *in vivo* is probably controlled by a number of as yet unknown mechanisms, and Gold, Reichow and co-workers (Gold et al., 2012; Kalman et al., 2006; Reichow et al., 2013) have shown that disrupting the interactions between AKAP2 (which acts as a scaffold for PKA) and AQP0 results in a cortical cataract, almost certainly because control of PKA to phosphorylation of AQP0 is disrupted. Moreover AQP0 gene knockout (Shiels et al., 2001) and a number of AQP0 mutations cause cataract (Francis et al., 2000; Hu et al., 2012; Okamura et al., 2003; Senthil Kumar et al., 2013; Wang et al., 2010a). AQP0 is not merely a solo player, but its interaction with other lens proteins (Gold et al., 2012; Lindsey Rose et al., 2006; Wang and Schey, 2011) is essential in lens homeostasis during development, differentiation and ageing (Kalman et al., 2006).

In the zebrafish lens, interference with the phosphorylation machinery, which modulates the calcium regulation of AQP0a water permeability results in a cataractous lens, providing more evidence that calcium regulation of AQP0 water permeability is essential for lens clarity (Clemens et al., 2013). The mounting evidence for both of the importance of calcium regulation of water permeability and the interaction of BFSP1 with AQP0 impelled us to test the effects of physiologically relevant C-terminal sequences from BFSP1 on the regulation of the water permeability of AQP0 by calcium. In particular, we felt it important to investigate the role of myristoylation and specific caspase cleavage as possible modes of AQP0 P_f_ regulation by calcium.

## 2. Material and methods

### 2.1. Sequence analysis software

The programs GrabCAS (Backes et al., 2005), CasCleave2 (Wang et al., 2014) (http://www.structbioinfor.org/cascleave2/) and MYRISTOYLATOR (Bologna et al., 2004) (http://web.expasy.org/cgi-bin/myristoylator/myristoylator.pl) were used to search for putative caspase sites and myristoylation motif in BFSP1. We used clustalW2 package (http://www.ebi.ac.uk/Tools/clustalw2/index.html) for sequence alignments and SeaView4.0 with default settings (Gouy et al., 2010) for its representation.

### 2.2. Cloning and purification of BFSP1 fragments

Full length human *BFSP1* was obtained from Source Bioscience (http://www.lifesciences.sourcebioscience.com/IMAGE clones 6154051 and 5406467). The C-terminal region of HsBFSP1 was amplified by PCR, the products cloned into and sequenced in pGEM-T Easy (Promega, UK; www.promega.com). Two caspase-resistant mutants, D433A and D549A BFSP1, a myristoylation defective (G434A) as well as domains within the C-terminal region of BFSP1 were generated by PCR and after sequencing were sub-cloned into pET28a vectors to generate protein by expression in BL21pLysS *E. coli* with a C-terminal histidine tag. Histidine tagged recombinant BFSP1 fragments were purified on Affinity Columns following manufacturers instructions (His-Select Nickel Affinity Gel; Sigma-Aldrich UK (www.sigmaaldrich.com)) and with a final purification step using a size exclusion column (Sephadex G10; Sigma-Aldrich).

### 2.3. In vitro caspase assay

Purified human recombinant BFSP1 and its D433A and G434A mutants (50 ng) were incubated with 5 units of purified recombinant activated caspases (1–10) for 3 h at 37 °C in caspase-assay buffer as recommended by the manufacturer (Enzo Life Sciences Technology, UK; www.enzolifesciences.com). BFSP1 and its fragments were then visualized with Coomassie Blue R250 staining after SDS-PAGE on 10% (w/v) SDS polyacrylamide gels, with modifications to the original published protocol (Laemmli, 1970), or using antibodies (3241; (Sandilands et al., 2004). His-Tag mouse monoclonal antibodies (Merck-Millipore #70796) were used to detect BFSP1 signal by immunoblotting after transfer of proteins from the gel to nitrocellulose paper using the semi-dry method (Kyhse-Andersen, 1984).

### 2.4. Myristoylation of BFSP1 at G434

The N-myristoyl transferase (NMT) substrates of the truncated BFSP1 Tail (GGQ-548P-His6) or PfARF1 (Plasmodium falciparum ADP-ribosylation factor 1) as the positive control were co-expressed separately with *Candida albicans* NMT (CaNMT) in BL21/pLysS *E. coli* strain in the presence of azido-myristate. CaNMT was subcloned into pET11c vector (ampicillin resistant) and co-transformed with protein substrates subcloned in pET28a (kanamycin resistant). Azido-myristoylated target proteins were captured and visualized by bioorthogonal labelling (CLICK Chemistry) as described previously (Heal et al., 2008, 2011). Briefly, after cell lysis and centrifugation, the soluble fraction was incubated with the appropriate capture reagent (Azido-TAMRA). Myristoylated proteins were separated by SDS-PAGE and detected with Cy3-TAMRA using a Typhoon 9400 fluorescence scanner (GE Health-care UK; http://www.gelifesciences.com/).

Km and Vmax values for human NMT1 and NMT2 using the octa-peptide (GGQISKGF) derived from HsBFSP1 were measured as described (Goncalves et al., 2012) and compared to values for the substrate standard, a pp60^src^ octapeptide, GSNKSKPK which is a demonstrated *in vivo* substrate (Lacal et al., 1988).

Values plotted are the mean from three assays made on the same day with the same proteins.

### 2.5. Cell culture and transient transfection

The mammary carcinoma cell line, MCF7 and the human lens epithelial cell line, FHL124 cells (Wormstone et al., 2000) were seeded in DMEM and EMEM respectively supplemented with glutamine, penicillin, streptomycin and 10% (v/v) FBS. Cells were transfected with constructs designed to express BFSP1 constructs that had been sub-cloned into pEGFPN3 expression vector (http://www.clontech.com/UK/Support/Applications/Using_Fluorescent_Proteins/EGFP_Vectors). Full length BFSP1 with or without the D433G and G434A mutations were transfected into MCF7 cells. For transfections into FHL124 cells, the 1–460 BFSP1 sequence fused to the N-terminus of eGFP was used to evidence cleavage at the D433 by caspase. Transfections were achieved using the GeneJuice transfection reagent (Novagen; http://www.emdmillipore.com/life-science-research/novagen/) according to manufacturers guidelines.

### 2.6. Caspase assays in MCF7 and FHL124 cells

MCF7 and FHL124 cells were transfected with wild type and caspase mutant BFSP1 fragments fused to the eGFP N-terminus. The caspase cleavage of BFSP1 was determined by immunoblotting to detect BFSP1 fragments using either the BFSP1 antibodies 3241 (Sandilands et al., 2004), or the eGFP antibodies (Cell Signaling Technologies, Rabbit polyclonal #2555). After transfection, MCF7 and FHL-124 cells were treated with 0.15 mM hydrogen peroxide for 3 h as an apoptogen to activate caspases. Cells were collected in lysis buffer (10 mM Tris-HCl pH6.8, 1 mM EDTA, 1X protease cOMPLETE inhibitor cocktail Roche (http://www.roche.co.uk), 1% (w/v) SDS) and then boiled after the addition of 5X sample buffer followed by SDS PAGE and immunoblotting.

### 2.7. Raising peptide antibodies against new neo-epitope sequences flanking the D433 caspase site

The short amino acid sequences (PLTQEGAPEDVPD: NP53 and PDGGQISKGFGKL: NPTail) flanking the D433 caspase site and myristoylation motif in HsBFSP1 were encoded into a nanoparticle carrier for immunization to prepare rabbit polyclonal antibodies following the described strategy (Schroeder et al., 2009) specific for caspase cleaved BFSP1 at site D433. Briefly, complimentary oligonucleotides were designed, annealed, phosphorylated and then cloned into the nanoparticle vector, and proteins were purified using the histidine tag in preparation for immunization and antibody production via a commercial source (Moravian Biotechnology (http://www.moravian-biotech.com). Pre-immune sera were checked for cross reactivity to recombinant HsBFSP1 before immunization. Sera were harvested after the 3rd boost.

### 2.8. Preparation of human eye lens extracts

Human eyes from donors in the age range 18–79 years were obtained from the Bristol Eye Bank with national research ethics committee approval and were used as recommended by the Declaration of Helsinki and following the procedures recommended under the Human Tissue Authority license to the University of Durham. Human lenses were dissected out from the eyes as soon as practically possible.

Lenses were decapsulated and isolation buffer (10 mM Sodium Phosphate pH7.4, 5 mM EDTA and 100 mM NaCl) was added in the ratio of 2ml/lens and stirred on ice for 15–20 min. This buffer to lens ratio was maintained for all subsequent extractions. The lens nucleus was removed and placed into fresh extraction buffer and stirred overnight at 4°C. Samples were homogenized with a Dounce homogenizer and centrifuged at 31,000×*g* @ r_max_ at 4°C for 20 min (Beckman SA20 rotor) to produce a pellet (P1) and supernatant (S1) fraction. This step was repeated. The plasma membrane cytoskeleton complex (PMCC) was prepared from S1 and S2 by recentrifugation at 80,000×*g* @ r_av_ at 4°C for 30min (Beckman SW28 rotor). To prepare the lipid membranes from the cortex and nucleus of the lens, the pellet fraction was then extracted sequentially with a series of buffers designed to remove all but integral membrane proteins. Each extraction was repeated once. The buffer series was as follows: 10 mM Sodium Phosphate pH7.4, 5 mM EDTA and 1.5 M KCl to generate pellets P3 and P4; 100 mM NH_4_HCO_3_ 1 mM EDTA; isolation buffer; 10 mM Sodium Phosphate pH7.4, 5 mM EDTA and 8 M urea; isolation buffer; 0.1 M NaOH; isolation buffer. The final lens membrane pellet was re-suspended in isolation buffer at a buffer ratio of 25μl/lens and stored at 4°C until required.

### 2.9. Immunogold labeling and scanning electron microscopy

Silicon mounts (Agar Scientific, UK) were cleaned with acetone and membrane samples were applied and incubated for 1 h at room temperature in a moist chamber. Mounts were fixed for 2 h in 2% (w/v) paraformaldehyde, 0.1 M cacodylate pH7.0, then transferred into PBS for 5min, then 100 mM glycine in PBS for 5min. Samples were blocked for 1 h in 1% (w/v) bovine serum albumin (BSA) in phosphate buffered saline (PBS) and incubated with primary antibody (1:50 dilution) for 60 min at room temperature in a moist chamber, then washed three times for 10min with PBS before incubating for 30 min at room temperature with the secondary gold-labelled antibodies (BBI Solutions; diluted 1:50). After washing in PBS, the mounts were left overnight at 4 °C in fix (0.1 M cacodylate buffer pH7.0, 2.5% (v/v) glutaraldehyde), then rinsed with 0.1 M sodium cacodylate pH 7.0 for 2min before post-fixing for 10min in 1% (v/v) osmium tetroxide in 0.1 M cacodylate pH7.0. They were dehydrated with a graded ethanol series before drying in a critical point dryer (Bal-Tech CPD 030) prior to coating with chromium (Cressington Scientific Instruments Ltd), ready for imaging in a JEOL 5400 SEM. Images from the scanning and backscattered electron modes for the instrument were collected and merged using Adobe Photoshop CS5.

### 2.10. cRNA preparation for Xenopus oocytes

The expression construct for bovine AQP0 (pXßG -AQP0) was a kind gift from Peter Agre (Baltimore MD). Wild type and mutated AQP0 cRNAs were transcribed *in vitro* using the T3 RNA polymerase (mMACHINE Kit; Ambion Inc.).

Expression constructs for different BFSP fragments were cloned from pET23 vectors with BamHI and BglII and ligating them into the BamHI cloning site in pXßG for expression in *Xenopus* oocytes. In most experiments 10 ng of cRNA of AQP0 and 10 ng of the experimentally tested BFSP fragment were injected into oocytes for expression. Typically two days elapsed between cRNA injection and measurement of P_f_ in the oocyte swelling assay.

### 2.11. Xenopus oocyte swelling assay

Oocytes were obtained in two ways. Female *Xenopus laevis* were anesthetized, and stage V and VI oocytes prepared and injected with 10 ng cRNA as previously described (Németh-Cahalan et al., 2004, 2007). In some experiments, especially later ones, oocytes were obtained from Ecocyte, Austin TX. The oocytes were incubated in 100% ND96 with the desired test Ca^2+^ concentration or pH for 5min before the swelling assay. Swelling assays were performed at room temperature (20–21 °C) by transferring oocytes from a 200 mOsm (100% ND96: 96 mM NaCl 96, 2 mM KCl, 5 mM HEPES, 1.8 mM CaCl2, 1 mM MgCl2, 2.5 mM sodium pyruvate, pH7.4) to a 70mOsm (30% (v/v) ND96) solution adjusted to the desired calcium concentration or pH. Water permeability, P_f_, was calculated from optical measurements of the increase in cross-sectional area of the oocyte with time in response to diluted ND96 using the formula:

$${\text{P}}_{f\;}\;=\;[d(\text{V}/V_{\text{o}})/d\text{t}][V_{\text{o}}/S]/[\text{Δ}o\text{s}m{\text{V}}_{W})]$$

where V is the volume as a function of time, V_0_ is the initial volume, S is the geometric surface area, Δosm is the osmotic gradient, and V_w_ is the molar volume of water. Error bars are ± SEM. A two-way ANOVA was performed with the software package R (www.r-project.org) to determine the statistical significance of the effect of calcium concentration, BFSP1 fragment, and their effect on AQP0 permeability. Each data point is the average of at least nine measurements (three different batches of oocytes, three oocytes from each batch). Error bars are shown as ± SEM. A two-way ANOVA was performed with the software package R (www.r-project.org) to determine the statistical significance of the effect of calcium concentration, BFSP1 fragment, and their interaction on AQP0 permeability.

### 2.12. Preparation of oocyte membrane protein and western blotting

Total membrane protein was isolated from *Xenopus laevis* oocytes using the ProteoExtract native membrane protein extraction kit (Calbiochem) according to the manufacturer’s directions. One oocytes worth of membrane protein in each lane was separated on a 4–12% SDS NuPAGE gel (Invitrogen) and transferred onto a nitrocellulose membrane. The blot was blocked in 5% (w/v) skimmed milk in Tris-buffered-saline with 0.1% (v/v) Tween (TBS-T), then incubated with anti-BFSP1 antibody (Rabbit polyclonal antibody 3241 (Sandilands et al., 2004);) diluted at 1:200 in blocking solution (1% (w/v) BSA, 2 mM EDTA in TBS-T). The immunoblot was then washed and incubated with goat anti-rabbit HRP conjugated antibody diluted at 1:1000 (Pierce) for 1 h at room temperature and visualized by ECL Prime Blotting Detection reagent (Amersham).

## 3. Results

### 3.1. Roadmap of results

Our results all lead to the conclusion that different BFSP1 fragments interact with AQP0 and modulate the regulation of its water permeability by calcium. We begin by showing that BFSP1 is a substrate for caspases and that caspase cleavage can generate C-terminal domain fragments of BFSP1 that alter AQP0 P_f_ regulation. We also demonstrate that myristoylation (which takes place at a cryptic site, G434, revealed by caspase cleavage) plays a critical role in the functional interaction of different BFSP1 fragments with AQP0. We show that AQP0 and BFSP1 fragments colocalize in the lens, and finally we demonstrate *in vitro* that the different BFSP1 fragments generated by caspase cleavage modulate calcium regulation of AQP0 P_f_ in different ways.

### 3.2. BFSP1 is a substrate for caspase cleavage and myristoylation

Proteomic data has suggested that BFSP1 is potentially cleaved by caspases and myristoylated (Wang et al., 2010b), but there are no biochemical data identifying the specificity of the caspases and NMTs for BFSP1.

**Fig. 1.**
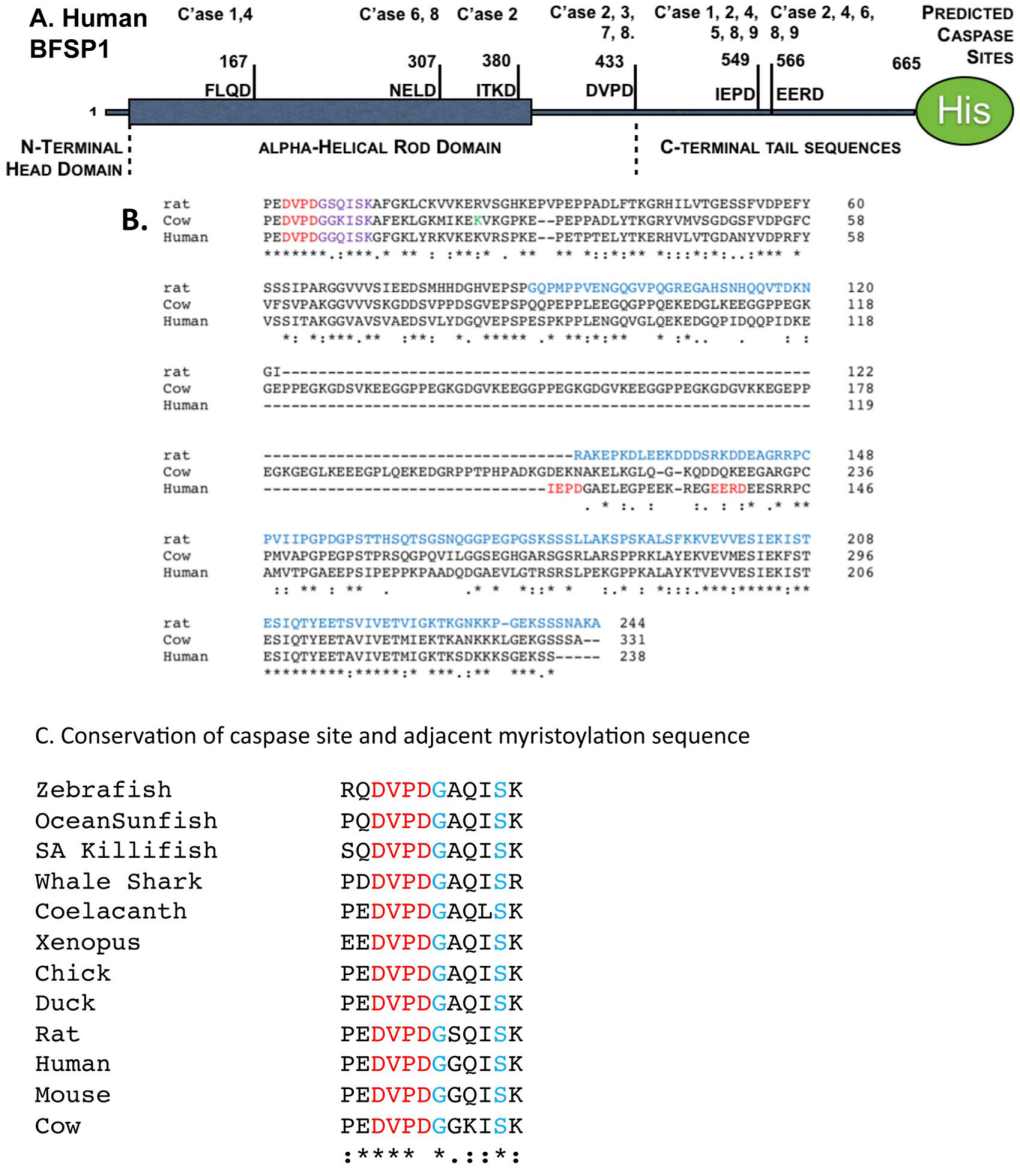
Bioinformatic analysis of the C-terminal sequences of BFSP1. **A**. The main structural features of human BFSP1 and the location of predicted caspase cleavage sites. The residue numbers for potential cleavage sites are included. **B**. Sequence alignment of human, rat and bovine BFSP1 using CLUSTALW to identify regions of homology. Caspase sites as predicted for BFSP1 are indicated (red). The caspase site adjacent to the cryptic myristoylation motif (mauve) is in a region of homology between human, rat and cow. The construct used to study the potential functional interaction of rat BFSP1 with AQP0 is written in blue script. The BFSP1 residue identified by crosslinking with AQP0 is indicated (green). Notice the relative positions of the myristoylation sequence and the predicted caspase sites and the variability in the location of these between the three species. **C**. The caspase site and adjacent cryptic myristoylation sequence in BFSP1 is conserved over a wide range of animals from whale shark to human.

We first analyzed whether BFSP1 was a potential caspase substrate in silico using the program CasCleave2 (Fig. 1A). Three potential sites were identified in the C-terminal region of BFSP1 (DVPD 430–433, IEPD, 446–449, and EERD, 566–569). If the site at D433 were cleaved, it would reveal a cryptic myristoylation motif. This was indicated by MYRISTOYLATOR and in agreement with the published proteomic data (Wang et al., 2010b). The C-terminal region of BFSP1 generally shows low sequence conservation between species (Song et al., 2009), but the caspase and associated myristoylation sequences (Fig. 1B) is a region that is highly conserved and adjacent to the K455 (Fig. 1B green label) which was identified by chemical cross-linking to interact with AQP0 (Wang and Schey, 2017).

The conservation of the caspase site (DVPD 430–433) and myristoylation motif (GGQISK, 434–439) is especially striking as seen by the alignment examples from cartilaginous (whale shark), ray-finned (killifish, sunfish, zebrafish) and lobe-finned fishes (coelacanth) as well as amphibians (*Xenopus*), birds (chicken, duck) and of course mammals (mouse, rat cow, human; Fig. 1C). This suggests that caspase cleavage and subsequent N-terminal myristoylation may have physiological significance for a very broad range of animals. The observed chemical cross-link between K455 in BFSP1 and D246 in AQP0 (Wang and Schey, 2017) also identifies this a region that interacts directly with AQP0. Indeed a C-terminal region of rat BFSP1 distal to D433 has been observed to increase AQP0 water channel activity about two fold, but the influence of myristoylation or the flanking sequences (Fig. 1B) on the regulation of AQP0 by BFSP1 was not considered (Nakazawa et al., 2011).

**Fig. 2.**
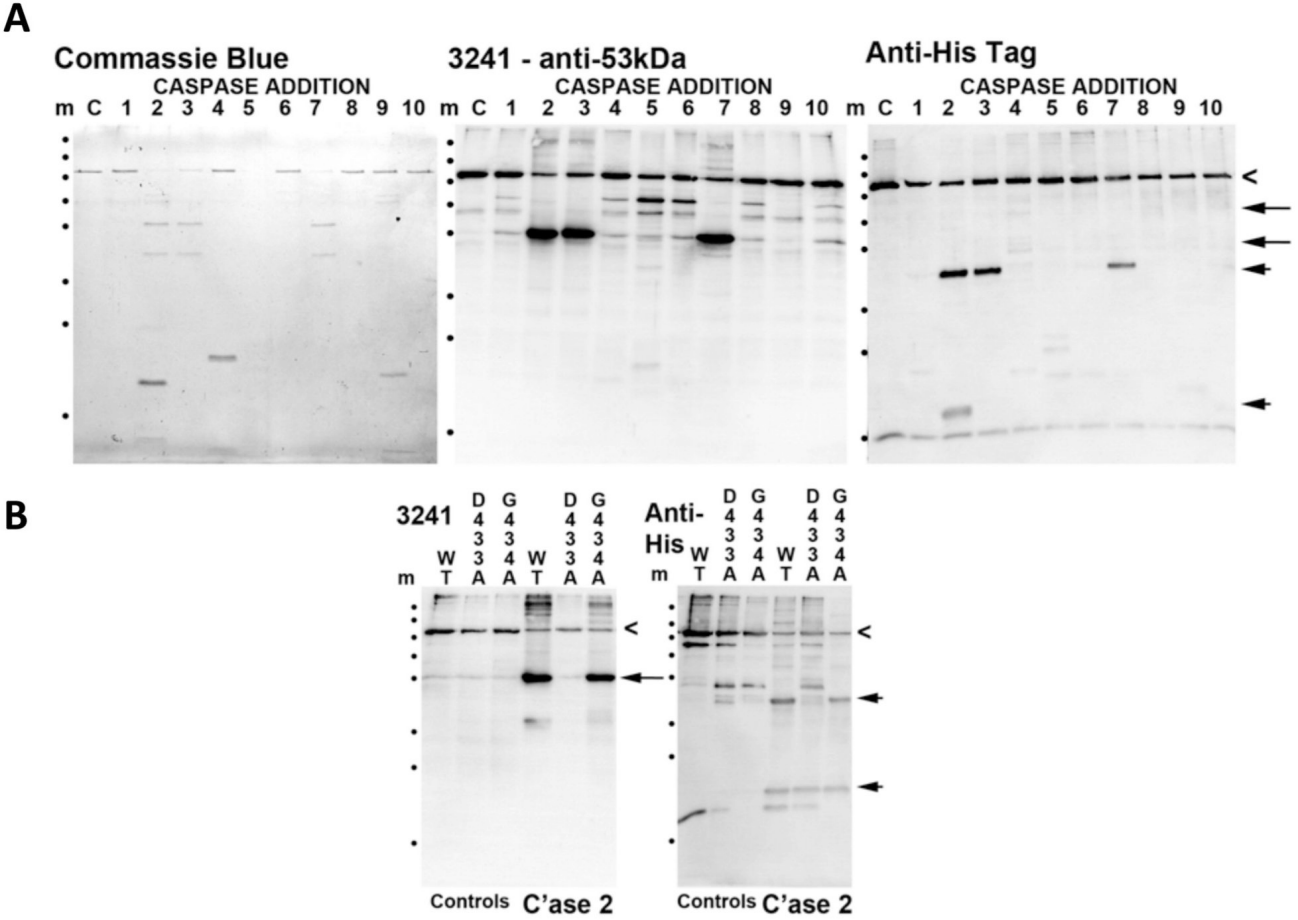
Characterization of the caspase sensitivity of BFSP1. **A**. *In vitro* cleavage of BFSP1 by purified caspases 1–10. Full length recombinant BFSP1 containing a C-terminal polyhistidine tag is indicated (chevron). Using the polyclonal antibody 3241 that was raised against the bovine 53 kDa fragment of BFSP1, two major fragments were detected (arrows). One corresponded to the 53 kDa fragment and like the slower migrating band of the two, both were undetected by the His-tag directed antibodies. Therefore both these fragments likely lack different proportions of the C-terminal sequences. Using the anti-His tag antibodies, two prominent bands were detected in the caspase 2 treated BFSP1 sample (arrowheads). Neither of these bands comigrated with bands detected by the 3241 polyclonal antibodies. **B**. To evidence cleavage of BFSP1 by caspase 2 at the D433 and D549 sites, two BFSP1 mutants were produced, D433A and D549A. Comparing WT, D433A and D549A BFSP1 sensitivity to caspase 2 cleavage resulted in the absence of the characteristic 53 kDa fragment (arrowhead) in the G433A BFSP1 as detected by the 3241 polyclonal antibodies. Using the anti-His tag antibodies, two characteristic fragments (arrowheads) were detected in the wild type and D549A BFSP1 samples, but only the faster migrating band in the D433A BFSP1 sample. Similarly the bands indicated (Asterisks) were present in the WT and D433A BFSP1 samples but missing from the D549A BFSP1 sample. This indicates that the mutations have abolished one site at position D433 and one at D549 for caspase 2.

Next we established biochemically that purified recombinant BFSP1 is indeed a substrate for activated caspases and identified those caspases specific for BFSP1 (Fig. 2A). Caspases 2, 3 and 5–7 all cleaved recombinant BFSP1 as demonstrated by immunoblotting using antibodies specific to the N-terminal region of BFSP1 (Fig. 2A; 3241) and to the C-terminal His-tag (Fig. 2A; Anti-His Tag). Notice that the fragmentation pattern for the two antibodies are different and there are also differences in these respective patterns after cleavage by the various caspases (Fig. 2A). We interpret the band detected by the 3241 antibodies as equivalent to a 53 kDa fragment ending at residue D433 (Fig. 1A), which is a major fragment produced after exposure to caspases 2, 3 and 7, all of which were proposed to cleave at D433 (Fig. 1A). To confirm this was indeed a caspase site, we disrupted the consensus sequence with the mutation D433A and found that this completely abolished the caspase 2 mediated cleavage of BFSP1 (Fig. 2). Mutating D549, another potential caspase 2 site, also altered the cleavage of BFSP1 (Fig. 2B, asterisks). We conclude that caspase 2 efficiently cleaves at residues D433 and D549 in the C-terminal domain of BFSP1.

**Fig. 3.**
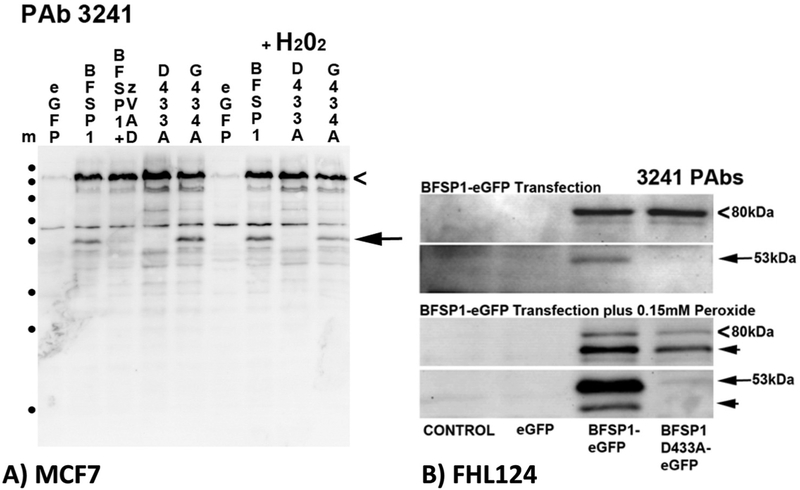
Caspase sensitivity of transfected BFSP1 in MCF7 and FHL124 cells. MCF7 and FHL124 cells were transiently transfected with constructs encoding eGFP alone, or as a N-terminal fusion with human BFSP1, and the mutants G433A (MCF7 and FHL124) and G434A BFSP1 (MCF7 only). Transfected BFSP1 was detected using the polyclonal antibodies 3241. **A**. MCF7 cells were either untreated or treated with the generic caspase inhibitor (zVAD) or with 0.15 M H202 in order to induce effector caspases. Full length eGFP-BFSP1 is indicated (chevron). A 53 kDa fragment (arrow) was detected by the 3241 antibodies. Note the absence of the 53 kDa signal in cells transfected with D433A, but not the G434A BFSP1 construct. The caspase inhibitor also prevented the formation of this fragment. **B**. The D433A mutation blocks the caspase sensitivity of BFSP1 constructs transiently transfected into the human lens epithelial cell line FHL124. The BFSP1 sequence 1–460 was fused to the N-terminus of eGFP and transiently transfected into FHL124 cells resulting in an 80 kDa product (chevrons). Notice the major breakdown product (53 kDa) for the construct containing the wild type D433 residue and the very significant reduction when this was replaced by A433. Slower migrating products (arrowheads) could arise by caspase cleavage either within the BFSP1 or eGFP parts of the fusion protein. Marker proteins (m) are indicated (•) and correspond to 250, 130, 100, 70, 55, 35, 25 and 15 kDa as per PageRuler prestained standards.

In order to demonstrate the caspase sensitivity of BFSP1 *in vivo*, BFSP1 constructs were transfected into two types of cultured cells (Fig. 3A and B). In both MCF7 cells (Fig. 3A) and the lens derived human cell line FHL124 (Fig. 3B), transiently transfected BFSP1 was also cleaved in a caspase dependent manner. The inclusion of the general caspase inhibitor zVAD prevented the detection by immunoblotting of discrete BFSP1 fragments in MCF7 cells (Fig. 3A). Introducing the D433A mutation also prevented the appearance of the 53 kDa BFSP1 fragment in transfected MCF7 cells (Fig. 3A, arrow) whilst the G434A mutation had little effect on the formation of the 53 kDa fragment (Fig. 3A, arrow). Similarly, in FHL124 cells, the D433A mutation prevented the appearance of a prominent 53 kDa product as identified by the 3241 antibodies (Fig. 3B, arrow) and several other products too (Fig. 3B arrowheads). The patterns achieved for the WT, D433A and G434A BFSP1 were not identical suggestive of a more complex caspase sensitivity for BFSP1 in MCF7 cells (Fig. 3A) and most likely FHL124 cells too (Fig. 3B) involving other BFSP1 caspase sites as predicted by our bioinformatic (Fig. 1A) and biochemical (Fig. 2A) analyses. These transient transfection data also support the conclusion that D433 is a *bone fide* caspase site in BFSP1 (Wang et al., 2010b).

**Fig. 4.**
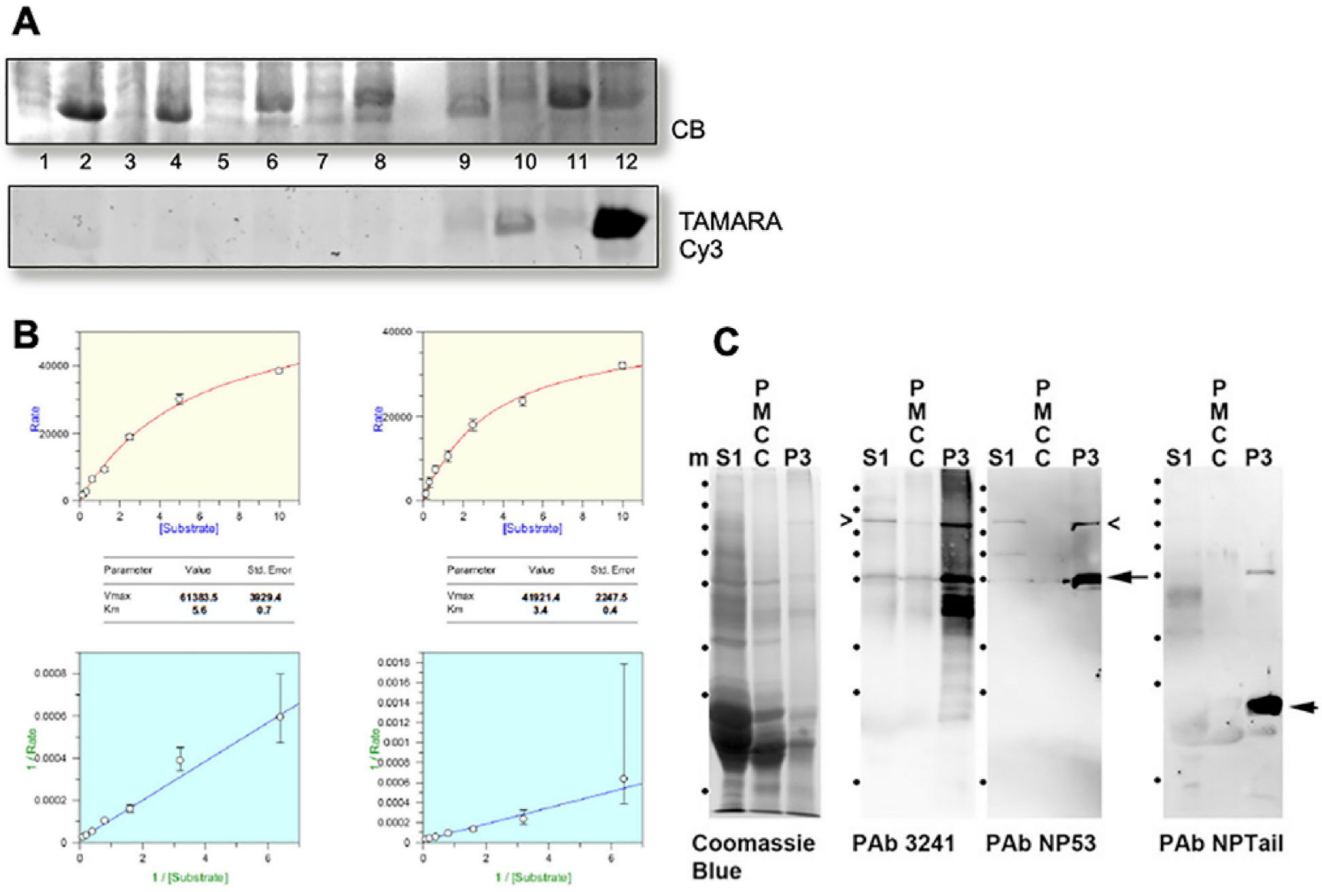
In vivo Myristoylation assay of truncated Tail fragment of BFSP1: **A**: Truncated Tail fragment of BFSP1 was co-expressed with CaNMT in *E. coli* in the presence of azido-myristate. CLICK-chemistry was used to detect myristolyated proteins and detected in the gel after fluorescently labelled with Cy3-TAMRA. PfARF1 (Plasmodium falciparum ADP-ribosylation factor-1) was included as a positive control of myristoylation. Track1 and 2, Pre- and post-induction of PfARF expression in *E.col*i; Tracks 3 and 4, Pre- and post-induction of PfARF with *C.albicans* NMT expression in *E.coli*; Tracks 5 and 6: Pre- and post-induction of G434-P548 BFSP1 expression in *E.coli*; Tracks 7 and 8, Pre- and post-induction of BFSP1 G434-P548 with *C.albicans* NMT expression in *E.coli*; Tracks 9 and 10, Pre- and post-induction of PfARF with *C.albicans* NMT expression in *E.coli* fed with azido-myristate followed by CLICK chemistry detection; Tracks 11 and 12, Pre- and post-induction of BFSP1 G434-P548 with *C.albicans* NMT expression in *E.coli* fed with azidomyristate followed by CLICK chemistry detection. Notice the positive bands in tracks 10 and 12 only. B. Km and Vmax determination for recombinant human NMT1 and 2 for the BFSP1 sequence G434- F441. C. Characterization of the polyclonal antibodies NP-Tail and NP53 using human lens samples. The soluble protein fraction (S1), the plasma membrane cytoskeleton complex (PMCC) and plasma membrane fraction (P3) were isolated from the human cortex and separated by SDS PAGE and the 10% (w/v) polyacrylamide gel prior to either staining with Coomassie Blue or immunoblotting and probing with the polyclonal antibodies 3241, NP-53 and NP-Tail. Notice that the major immunoreactive band detected in the P3 sample by the NP-tail antibodies would correspond to a C-terminal fragment of less than 25 kDa. Marker proteins (m) are indicated (•) and correspond to 250, 130, 100, 70, 55, 35, 25 and 15 kDa as per PageRuler prestained standards.

To demonstrate the myristoylation potential of the N-terminal G434 in the caspase-cleaved BFSP1, we took two different approaches. In the first, a bacterial system that co-expressed the *Candida albicans* NMT with a putative target was analyzed (Fig. 4A). Only when both azidomyristate and the NMT were present in the same bacterial extract was a positive signal detected (Fig. 4A, tracks 10 and 12) for the positive control (PfARF1) and for the BFSP1 C-terminal sequences starting at G434. In the second approach (Fig. 4B), the BFSP1 octapeptide spanning residues 434–441, GGQISKGF, was analyzed for its substrate potential using recombinant human NMT1 and NMT2. The Km (5.7 ± 0.7 μM and 3.4 ± 0.7 μM for NMT1 and NMT2 respectively; Supplementary Fig. 1) values obtained for the BFSP1 octapeptide were comparable to those obtained for the pp60^src^ octapeptide standard(2.5 ± 0.2 and 0.9 ± 0.1 μM μM for NMT1 and NMT2 respectively) for as reported previously for this substrate *in vitro* assay (Goncalves et al., 2012). NMT1 and NMT2 are both present in the lens (Shrivastav et al., 2004). These data therefore provide the biochemical evidence to support the previous proteomic data that identified a myristoylated G434 after cleavage at D433 (Bassnett et al., 2009; Wang et al., 2010b, 2013; Wang and Schey, 2015).

Azido-myristate very efficiently labelled many proteins in MCF7 transfected cells (data not shown) making it difficult to detect cleaved myristoylated BFSP1. We therefore generated two polyclonal antibodies (NP53 and NPtail) to identify the DVPD and GGQSIK containing epitopes in BFSP1. We demonstrate that these two antibodies identify different immunoreactive banding patterns in samples from human lens plasma membranes (Fig. 4C). Indeed the major fragment detected by the NPtail antibodies in alkali stripped, lens fiber cell plasma membranes was less than 25 kDa indicating that the C-terminal region is not only cleaved at position D433, but also very likely at other distal sites as predicted (Fig. 1A).

### 3.3. Co-immunolocalization of BFSP1 C-terminal sequences and AQP0 in human lens plasma membranes

**Fig. 5.**
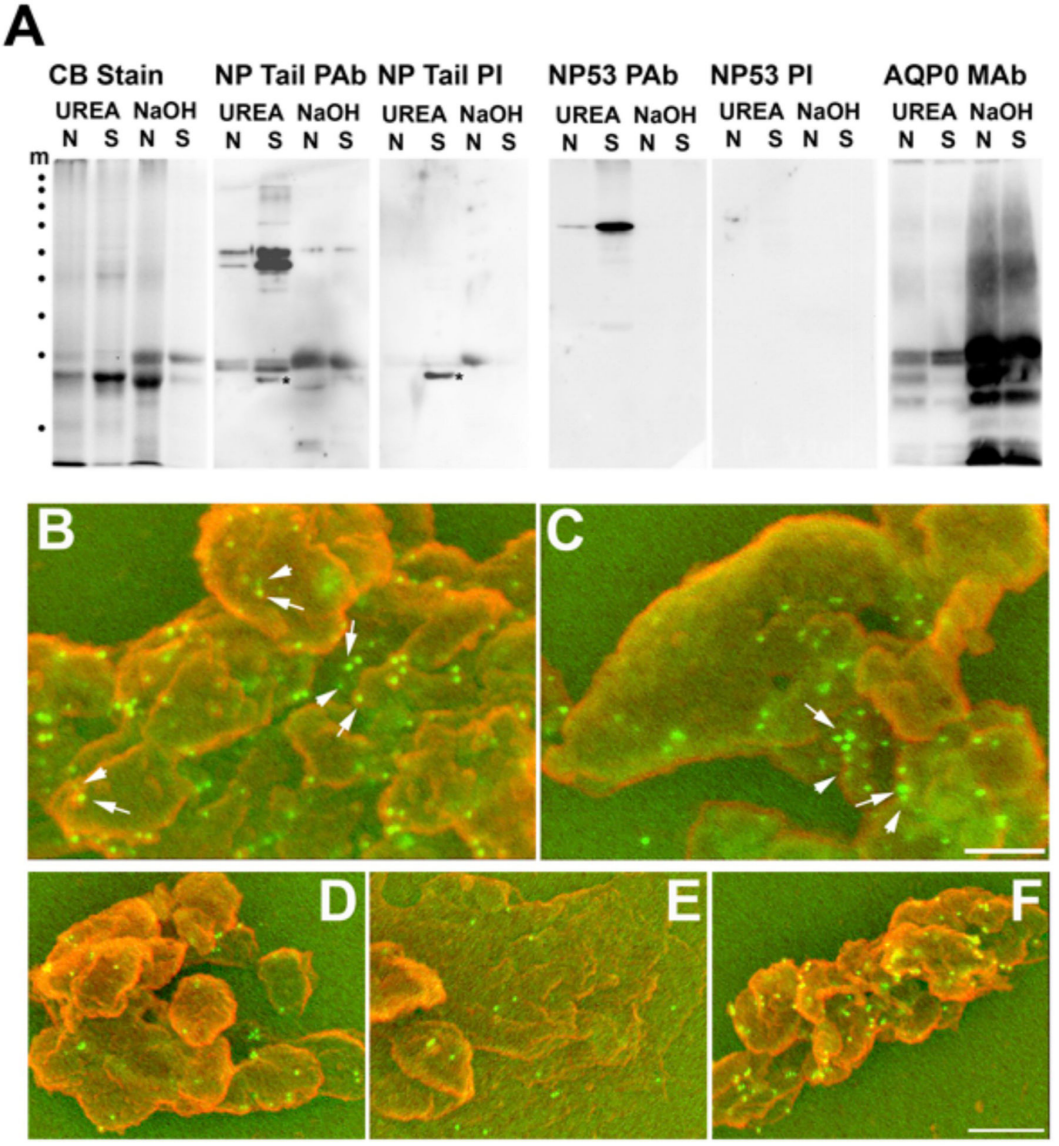
Cofractionation and colocalization of BFSP1 and AQP0 to the plasma membrane compartment of lens fibre cells. **A**. Plasma membranes were prepared from the nucleus (N) and cortical (C) parts of the lens from a 51 year old male donor. The plasma membranes were extracted with buffer containing 8 M urea (UREA) and then with 0.1 M NaOH (NaOH) and samples of the pelleted material taken and separated by SDS PAGE. Coomassie blue staining of the proteins in these fractions (CB Stain) revealed a few prominent low molecular weight proteins between the 25 and 15 kDa markers. Immunoblotting of these plasma membrane fractions with the polyclonal antibodies NP-53 identified BFSP1 N-terminal-derived fragments only in the urea extracted membranes. These were removed after alkali extraction. In contrast, the NP Tail antibodies identified bands in both urea and alkali extracted membrane fractions, although the fragments retained in the alkali stripped membrane fraction were those with faster electrophoretic mobility equivalent to approx. 25 kDa. Probing these fractions with AQP0 antibodies demonstrated the enrichment of AQP0 signal in the alkali stripped plasma membrane fractions of both the nuclear and cortical parts of the lens. Marker proteins (m) are indicated (•) and correspond to 250, 130, 100, 70, 55, 35, 25 and 15 kDa as per PageRuler prestained standards. **B, C**. Co-immuno-gold labeling of alkali stripped plasma membranes from the nucleus (B) and cortex (C) of human lenses using the rabbit polyclonal NP Tail antibodies to detect BFSP1 and mouse monoclonal antibodies to detect AQP0. To visualize the antibodies in the SEM, 5 nm and 10 nm gold labelled secondary antibodies were used to detect AQP0 (arrowheads) and BFSP1(arrows) respectively. **D, E and F**. Immunogold labeling of alkali stripped membranes from human lens nuclei and labelled with either the preimmune serum for the NP Tail antibodies (D) or the polyclonal antibodies NP-53 (E). These two evidence the background labeling achievable either with preimmune (D) or closely related polyclonal rabbit antibodies (E). The NP53 detectable BFSP1 fragments have been removed by the alkali extraction of these membrane preparations (A). The signal detected by the polyclonal NP Tail antibodies (F) is very clearly above background levels.

Previous biochemical fractionation of the lens identified a ~51 kDa C-terminal BFSP1 fragment associated with lens plasma membranes even after urea extraction (Brunkener and Georgatos, 1992). It was important to show that the BFSP1 C-terminal region distal to G434 was indeed a resident plasma membrane polypeptide and to confirm recent proteomic analyses (Bassnett et al., 2009; Wang et al., 2010b, 2013; Wang and Schey, 2015), we utilized the NPTail and NP53 polyclonal antibodies to identify both the caspase cleavage site (NP53 PAb) and the myristoylation sequence (NPTail PAb) in samples prepared from a single male 51 year old donor. Both sera detected complex patterns in human lens extracts, which were absent from the pre-immune sera (Fig. 5A). We confirmed that alkali-extracted plasma membranes purified from the cortex and nucleus of human lenses retained only the C-terminal sequences containing the myristoylation sequence in donor lenses aged from 18 to 79 and in both the nuclear and cortical lens membranes (Supplementary Fig. 2). Moreover, these alkali stripped membranes were enriched in BFSP1 fragments in the 20 kDa range immunoreactive with these antibodies indicating that further proteolysis had likely occurred (Fig. 5A; Supplementary Fig. 1). Using the NP53 polyclonal antibodies to detect the adjacent caspase-cleavage site to the myristoylation sequence, we found that this N-terminal portion of BFSP1 was lost from the alkali-stripped membranes (Fig. 5A). Immunoblotting of the same fractions with AQP0 antibodies confirmed that the fractionation enriched for integral membrane proteins as they were retained after alkali stripping (Fig. 5A).

We then used these antibodies to immunolocalise both BFSP1 C-terminal sequences containing the myristoylation motif and AQP0 on alkali stripped plasma membranes prepared from the 51 year old human lens nucleus (Fig. 5B) and cortex (Fig. 5C) by scanning electron microscopy. Previous immunofluorescence analysis had shown by fluorescence light microscopy that both proteins occupy the same plasma membrane regions in the lens (Lindsey Rose et al., 2006), but immunogold electron microscopy provides greater resolution. Whilst NP53 antibodies (Fig. 5E) and preimmune serum (Fig. 5D) gave very little signal, the NP Tail serum clearly stained the lens membranes (Fig. 5F) Co-staining of plasma membranes with both NPTail and B11 antibodies (Fig. 5B and C; BFSP1, 10 nm gold particles; AQP0, 5 nm gold particles) showed that AQP0 and the G434 containing fragments of BFSP1were adjacent to each other in the membrane preparations (Fig. 5B and C; arrows and arrowheads). These data indicate that AQP0 and BFSP1 C-terminal fragments containing the myristoylation sequence are in very close proximity at the plasma membranes of the human lens. These data are in agreement with previous crosslinking (Wang and Schey, 2017) and proteomic (Bassnett et al., 2009; Wang et al., 2010b, 2013; Wang and Schey, 2015) data.

### 3.4. Different fragments of BFSP affect AQP0 regulation differently

**Fig. 6.**
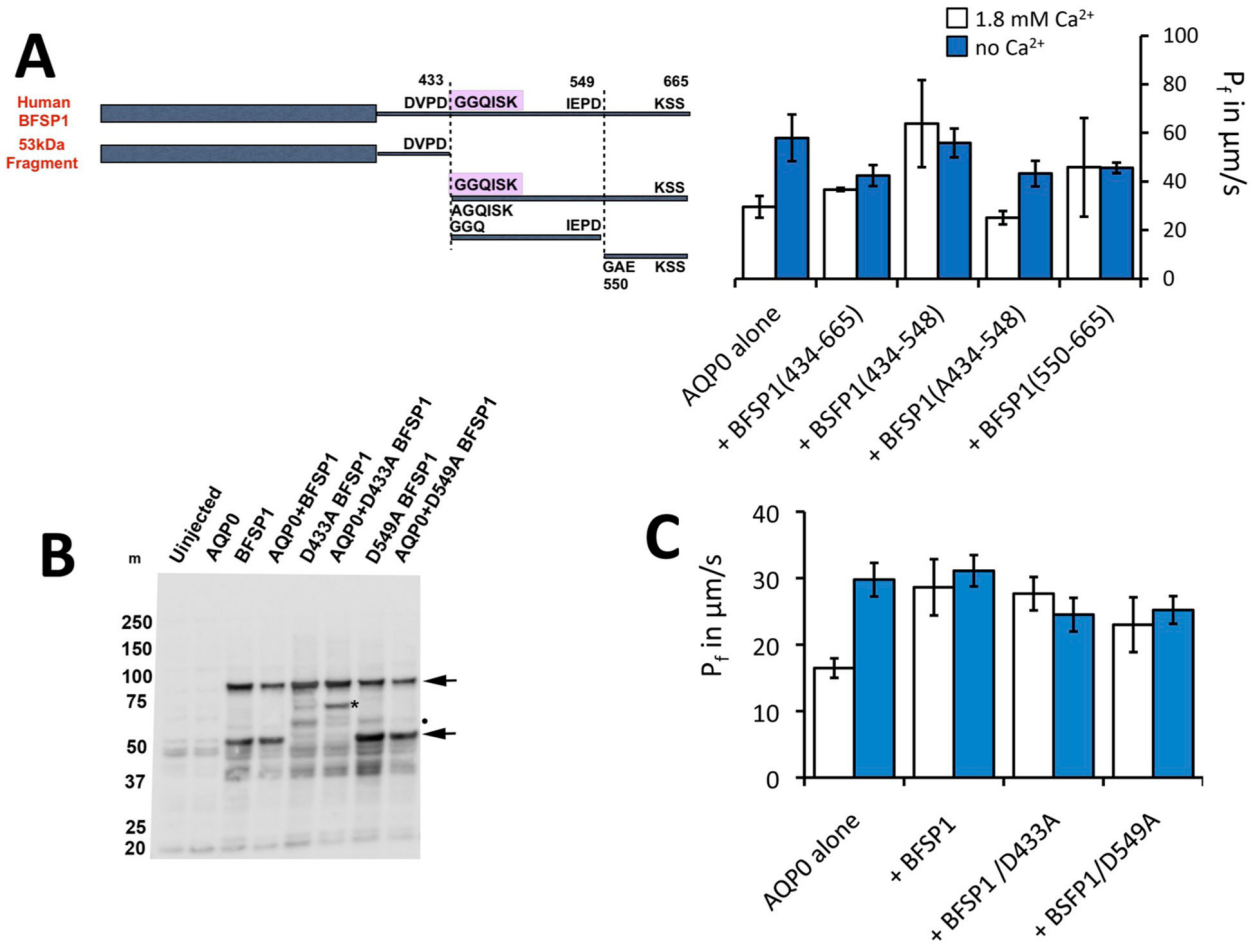
Regulation of AQP0 by BFSP1 C-terminal domains. **A**. *Xenopus* oocytes were injected with AQP0 alone or in combination with the indicated BFSP1 constructs. C-terminal constructs could be divided into two classes: ones which left the calcium response essentially identical to wild type, that is P_f_ was low in 1.8 mM Ca^2+^ and high in 0 mM Ca^2+^; or ones in which P_f_ did not change with Ca^2+^ concentration. Results were analyzed by omnibus ANOVA followed by pair wise t-tests for individual 0 mM Ca^2+^ - 1.8 mM Ca^2+^ comparisons. In the cases where P_f_ increased in response to no calcium, pairwise p values were less than 0.05 (*). In cases where P_f_ did not respond to changes in calcium, p values were greater than 0.3 (ns). Conditional p values for 1.8 mM Ca^2+^ vs no Ca^2+^: AQP0 alone = 0.0232, +434–665 = 0.0328, +434–548 = 0.6823, +A434–548 = 0.0128, **B**. Immunoblots showing that BFSP1 was expressed in *Xenopus* oocytes when appropriate constructs were injected. Two major immunoreactive bands were detected (arrows), the faster migrating band a fragment derived by proteolytic cleavage because the D433E mutation in BFSP1 altered the pattern achieved. Notice too that the presence of AQP0 altered the banding pattern achieved with the D433A mutant (*). BFSP1 immunoreactive bands are found in the pellet fraction prepared from injected oocytes. **C**. Full length BFSP1 and full length BFSP1 with mutations at putative caspase sites eliminated P_f_ regulation by calcium. Conditional p values for 1.8 mM Ca^2+^ vs no Ca^2+^: AQP0 alone = 0.001, +BFSP1 = 0.617, +BFSP1 D433A = 0.3913, +BFSP1 D549A = 0.6445.

Our caspase sensitivity (Figs. 2 and 3) and immunoblotting (Fig. 5A) data suggest that the BFSP1 C-terminal sequence distal to the caspase-cleavage site at D433 is likely further processed by proteolysis. Evidence for such additional caspase-mediated proteolysis is not apparent in the literature, but identifying such sites will require further investigation (Wang and Schey, 2011). We designed a series of constructs (Fig. 6A) to investigate the potential roles of the myristoylation motif in BFSP1 and other C-terminal sequences based on the predicted caspase sites at D433 and D549 (Figs. 1 and 6A). Although we have no evidence as yet to suggest that D549 is a physiological caspase site, it provided a rationale to divide the C-terminal region of BFSP1 into smaller domains in order to probe potential functional differences. Immunoblots showed that BFSP1 was expressed and located in the membrane fraction prepared from injected *Xenopus* oocytes (Fig. 6B).

Most of the BFSP1 C-terminal fragments, full length BFSP and full length BFSP containing the D433A mutant and the D549A mutant lock the water permeability of AQP0 high and eliminate the increase of P_f_ in response to low Ca^2+^ (Fig. 6A and Table 1). The fragment, A434 – P548, has no effect on the response of AQP0 to calcium. (See Fig. 6A and Table 1). The G434 – S665 fragment blunts the calcium response even though the difference between P_f_ in 0 mM Ca^2+^ and 1.8 mM Ca^2+^ is marginally significant.

For wild type (WT) AQP0 alone, 0 mM Ca^2+^ approximately doubles P f. But if serine 235 is phosphorylated, 0 mM Ca^2+^ has no effect on P_f_. The data in Fig. 6A and Table 1 establish that fragments which contain a competent myristoylation site at D434 are capable of altering the Ca^2+^ regulation of P_f_ and, with the probable exception of the G434 – S665 fragment, of locking the P_f_ of AQP0 high.

We also evaluated the effects of full length BFSP1 and full length BFSP1 with a mutation at caspase site 433 or 549 (D433A or D549A; Fig. 6B). Notice the presence of a major immunoreactive band in addition to the full length BFSP1 (Fig. 6B; arrowheads), which was eliminated by the D433A mutation in BFSP1. Notice too that the immunoreactive banding pattern was affected by co-transfection with AQP0 (Fig. 6B D433A BFSP1 cf AQP0+D433A BFSP1; asterix). A second caspase cleaved product (Fig. 6B D459A BFSP1 and AQP0+D459A BFSP1; dot) was retained in both the D433A and D549A mutants, which was absent from the wild type BFSP1 sample. Full length BFSP1 and full length BFSP1 with either caspase-nulling mutation both eliminated P_f_ regulation by altered Ca^2+^ concentration (Fig. 6C).

## 4. Discussion

**Fig. 7.**
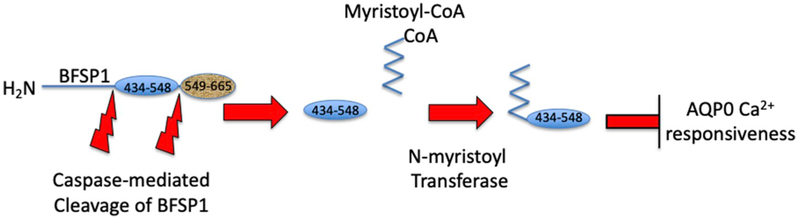
AQP0 activity is differentially modulated by BFSP1 and its post-translationally modified fragments. Full length human BFSP1 is cleaved by caspases at sites 433 and 549. The 433 site reveals a cryptic myristoylation site. The lens expresses both NMT1 and NMT2. The 434–549 and 434–665 fragments, once myristoylated, can effectively eliminate the regulation of AQP0 P_f_ by Ca^2+^. Preventing myristoylation restores the Ca^2+^ regulation of AQP0 P_f_ (right hand side of the figure).

In this paper we provide biochemical evidence to support the observed myristoylation of BFSP1 (Wang et al., 2010b) at a cryptic C-terminal site (G434) revealed after caspase cleavage. We show that C-terminal tail fragments derived from full length BFSP1 by caspase cleavage can affect the calcium regulation of the water permeability of AQP0 using a *Xenopus* oocyte swelling assay. We also show that blocking the myristoylation potential of such fragments can reverse this potential regulation (Fig. 7). Whilst direct translation to lens fiber cells is speculative, the data suggest important physiological implications for the calcium mediated effects upon AQP0-dependent water transport in lens fiber cells.

### 4.1. BFSP1 – a substrate for both caspase and NMT activities

A myristoylated fragment of BFSP1 was previously identified in the bovine lens (Wang et al., 2010b) and we have demonstrated *in vitro* that the sequence GGQISK is efficiently myristoylated *in vitro* by both human NMT1 and 2 and when co-tranfected with a fungal NMT in *E. coli*. The eye lens contains both NMT1 and 2 (Shrivastav et al., 2004) that actively myristoylate a specific subset of lens proteins (Cenedella and Chandrasekher, 1999). The myristoylation motif in BFSP1 is a cryptic site requiring protein cleavage for it to become available for myristoylation. Caspase activity is readily detected in the absence of apoptotic signals in the lens (Foley et al., 2004; Weber and Menko, 2005). The BFSP1 myristoylation motif is preceded immediately by a conventional caspase consensus site (Fig. 1) that is readily cleaved *in vitro* and in MCF7 cells (Fig. 2) and in lens derived human FHL124 cells (Fig. 3B). We suggest that the caspase-mediated cleavage to expose a cryptic myristoylation site is a credible mechanism to explain the observed myristoylation of BFSP1 in lens membrane fractions (Wang et al., 2010b). Our data confirm that BFSP1 is a substrate for multiple caspases (Fig. 2) and we have demonstrated that BFSP1 is cleaved at D433 (Figs. 2 and 3) in tissue culture cells, including the lens epithelial cell line FHL124 (Fig. 3). Whilst these data do not identify which caspase(s) might be responsible, the lens clearly has caspase and caspase-like activities (Foley et al., 2004; Weber and Menko, 2005; Zandy and Bassnett, 2007) that would expose the cryptic myristoylation site in BFSP1. Indeed, the site (DxxDG) in BFSP1 conforms to the most favored to effect caspase cleavage (Mahrus et al., 2008). It is worth noting that the aspartate residue D433 in the caspase recognition sequence, undergoes isomerization (Wang et al., 2010b). This post-translational modification is residue specific as other potential DG sites remain unchanged in BFSP1. Moreover the modification is found only in uncleaved peptides (Wang et al., 2010b), suggesting this might modulate caspase cleavage at D433 in BFSP1 and introducing a further mechanism to alter its interaction with AQP0.

There are also many examples where post-translational myristoylation is associated with the exposure of a cryptic site after caspase cleavage of the protein (reviewed in (Martin et al., 2011)). We suggest that BFSP1, like actin (Utsumi et al., 2003), gelsolin (Sakurai and Utsumi, 2006) and cytoplasmic dynein intermediate chain 2A (Martin et al., 2012) and regulators of the cytoskeleton (Block et al., 2012; Han et al., 2009; Martin et al., 2012; Ott et al., 2013; Vilas et al., 2006) is another instance where the exposure of a cryptic myristoylation site after protein cleavage is a regulatory mechanism. Whilst many of these mechanisms are linked to apoptosis some have been found to be linked to differentiation such as PAK2 (Cathelin et al., 2006). In the case of gelsolin (Sakurai and Utsumi, 2006) and ctPKCε (Martin et al., 2012), caspase cleavage and myristoylation protects against apoptosis. Caspase activity is also required during development (Busso et al., 2010) and metastatic cell invasion (Rudrapatna et al., 2013).

### 4.2. Functional role of BFSP1 myristoylation

Myristoylation is insufficient to tether a protein to a membrane (Peitzsch and McLaughlin, 1993), but requires other proteins or poly-basic sequences to effect membrane targeting (Resh, 2004). For this reason myristoylation is believed to be a regulatory post-translational modification (Martin et al., 2011; Resh, 2006; Zha et al., 2000). Analysis of the C-terminal sequences of BFSP1 (Fig. 1A) revealed that the myristoylation site was indeed conserved from whale shark to mammals (Fig. 1C) and was adjacent to a lysine/arginine-rich region. Following BFSP1 cleavage by caspase activities, the fragments derived from this C-terminal region of BFSP1 resisted urea and alkali extraction (Figs. 4C and 5A) in agreement with previous proteomic analysis of such fractions from human lens fibre cell plasma membranes (Wang et al., 2010b, 2013; Wang and Schey, 2015). This evidences that they are strongly bound to the plasma membrane with properties more similar to integral membrane proteins like eg gap junction proteins (Milks et al., 1988). AQP0 bound BFSP1 in an affinity chromatography approach (Lindsey Rose et al., 2006) and recently a chemically induced cross-link between the D246 in AQP0 and K455 in BFSP1 was also reported (Wang and Schey, 2017). Our immunoelectron microscopy data (Fig. 5) confirm that AQP0 and the C-terminal fragment of BFSP1 containing the myristoylation sequence are in very close proximity to each other in alkali extracted membrane preparations of human lenses. An arbitrarily selected C-terminal fragment was reported to potentially regulate AQP0 water channel activity (Nakazawa et al., 2011). Our data now assess whether AQP0 can potentially be regulated by caspase generated BFSP1 fragments in a myristoylation-dependent manner.

We report here that the myristoylation competent fragment G434-S665 eliminates the calcium regulation of AQP0 (Fig. 6; Table 1). Our attempt to inhibit myristoylation pharmacologically in the *Xenopus* oocyte system was unsuccessful as AQP0 activity was completely inhibited by the inhibitor (data not shown (Rackham et al., 2013);). However, comparison of the effects of two nearly identical fragments, A434 – P548 and G434 – P548, clearly demonstrates that myristoylation could play a critical regulatory role. The myristoylation competent fragment, G434 – P548, locks P_f_ high and eliminates the low Ca^2+^ increase in P_f_. The nearly identical, but myristoylation incompetent fragment, A434–P548, however, does not alter the ability of low calcium to increase AQP0 P_f_. Thus myristoylation of this fragment is sufficient to eliminate the calcium regulation of AQP0 P_f_.

Our data also indicate that the 434–665 sequences of human BFSP1 potentially have multiple caspase sites capable of producing BFSP1 fragments to AQP0 water channel activity, but further investigations are needed to identify those that are physiologically important in the lens.

The simplest interpretation of our data (Fig. 6B,C) is that caspase 2, present in *Xenopus* oocytes (Nutt et al., 2009), generates BFSP1 C-terminal fragments that interact directly with the coinjected AQP0 (Wang and Schey, 2017). We know from measuring the effects of particular fragments on AQP0 P_f_ regulation that some will alter the calcium sensitivity of AQP0 P_f_ in a myristoylation dependent manner. One or more of these fragments would be generated from full length BFSP1 if caspases were present, and this accounts for the ability of full length BFSP1 containing the mutants D433A and D549A to alter the calcium sensitivity of AQP0 in the *Xenopus* oocyte assay.

A previous study arbitrarily selected a BFSP1 C-terminal sequence to investigate potential effects upon AQP0 water permeability (Nakazawa et al., 2011) without considering potential caspase site location or subsequent myristoylation (Fig. 1A). The present study shows that the presence of caspase sites and the availability of a cryptic myristoylation site can all influence the water permeability of AQP0 by altering the sensitivity of AQP0 P_f_ to calcium (Fig. 6). All the experimental conditions except the test osmotic solution are identical, so neither change in expression nor changes in transport to the membrane can account for the altered regulation we have observed. This is contrast to a previous study (Nakazawa et al., 2011) where the magnitude of the P_f_ is the sole quantity measured and where the potential role of myristoylation was not considered.

### 4.3. Direct or indirect regulation of AQP0 by BFSP1 C-terminal domains

A potential question is whether the regulation of AQP0 by BFSP1 is mediated directly or indirectly. The latter could be by, for instance, an interaction of BFSP1 fragments with another protein that in turn interacts with AQP0, such as AKAP2 (Gold et al., 2012). There is no evidence to date that BFSP1 fragments interact with calmodulin or AKAPs, although another intermediate filament protein, synemin, is reported to be a cardiac AKAP (Russell et al., 2006). PKA is an important regulator of AQP0 via phosphorylation (Gold et al., 2012) and the plasma membrane, urea/alkali resistant BFSP1 is also phosphorylated at S488 as reported in the recent proteome of lens membrane proteins (Wang et al., 2013), but this is not a consensus PKA site (http://kinasephos2.mbc.nctu.edu.tw/predict.php) that could suggest an avenue for future investigation given the report of a chemical cross-link between D246 in AQP0 and K455 in BFSP1 (Wang and Schey, 2017). The myristoyl group (G434–548 versus G434A-548) could potentially attenuate the regulation of AQP0 in the lens after caspase cleavage at D433 via the lipid environment of AQP0 itself (Tong et al., 2013) and interestingly AQP0 is also acylated at lysine 238 with oleic acid (Schey et al., 2010). Coincidentally K238 is near the BFSP1 binding region and its myristoylation sequence.

### 4.4. Summary

Even though we do not know the detailed mechanism of AQP0 P_f_ regulation, our data clearly show that it can be modulated by C-terminal domain fragments of BFSP1 generated by caspase cleavage (Summarized in Fig. 7). Moreover, the revelation of a cryptic myristoylation site generates a fragment (G434–548) with the ability to modulate AQP0 P_f_ in the presence of calcium while a nearly identical fragment (G434A-548) lacks this ability. AQP0 is essential for lens clarity and proper development. Mutant Aqp0a lacking P_f_ regulation by Ca^2+^ cannot rescue knockdown of wild type Aqp0a in zebrafish (Clemens et al., 2013). We conjecture the modulation of AQP0 P_f_ is essential for proper lens development and for proper generation of the appropriate gradient of index of refraction (Vorontsova, I., Gehring, I., Hall, J. E., & Schilling, T. F. (2018). Aqp0a Regulates Suture Stability in the Zebrafish Lens. *Investigative Ophthalmology & Visual Science*, *59*(7), 2869–2879. http://doi.org/10.1167/iovs.18-24044). Clearly BFSP1 plays a role in this modulation. The details of when and where in the lens this regulation takes place remain to be worked out and will be the subject of future investigation.

## Supplementary Material

1

2

3

4

## Figures and Tables

**Table 1:** Effects of BFSP1 Fragments on AQP0 calcium regulation.

BFSP1 fragments		no effect on AQPO calcium regulation	Locked high AQPO calcium regulation
Full length			X
Full length/D433A	disrupts myristoylation site		X
Full length/D549A	disrupts one caspase site		X
434–548	myristoylation site, two LBDs		X
A434–548	no myristoylation site	X	
434–460	one LBD	X	
461–548	one LBD	X	
550–665	no myristoylation site, two LBDs		X

## Abbreviations:

AQP0: Aquaporin zero
BFSP: Beaded filament structural protein
EPPD: Ezrin, Periplakin, Periaxin, and Desmoyokin)
BFSP1 HsBFSP1: BFSP2
AQP0: Aquaporin zero
AKAP: AKAP2 A-Kinase Anchor Proteins
PKA: Protein Kinase A
DMEM: Dulbecco’s Modified Eagle Medium
EMEM: Eagle’s Minimum Essential Medium
MCF7: Breast cancer cell line named after Michigan Cancer Foundation (MCF)
FHL124: Fetal Human lens cell line 124; eGFP, enhanced Green Fluorescent Protein
NMT1: N-myristoyltransferase 1
NMT2: N-myristoyltransferase 2
CaNMT: Candida albicans myristoyl-CoA: protein N-myristoyltransferase
EDC: 1-Ethyl-3-(3-dimethylaminopropyl) carbodiimide, a crosslinker
