# Supplementary figures and images for: BFSP1 C-terminal domains released by post-translational processing events can alter significantly the calcium regulation of AQP0 water permeability
Source: Exp Eye Res. Author manuscript; Available in PMC 2020 Aug 1. (PMC6713518; doi:10.1016/j.exer.2019.02.001)

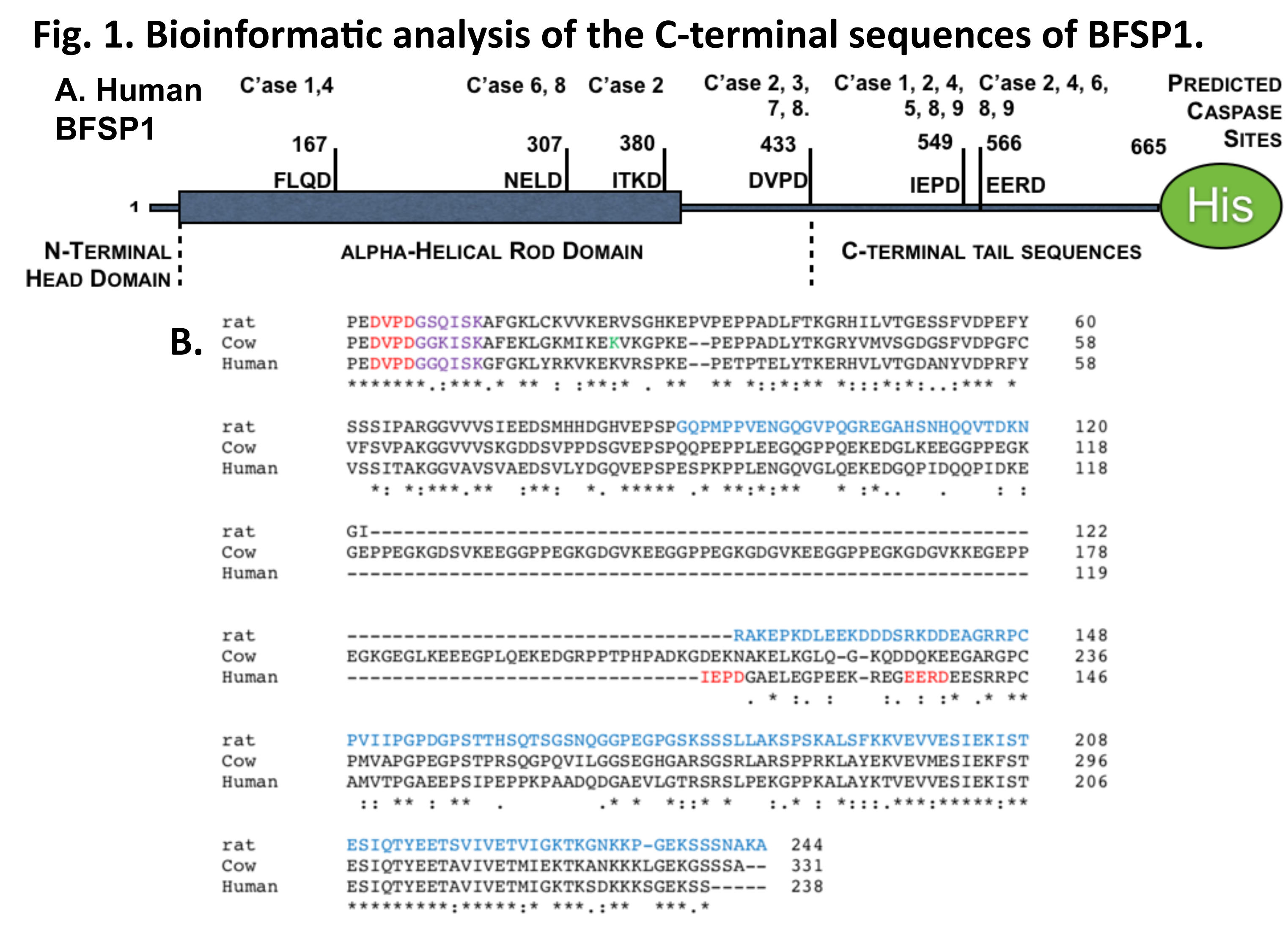

Supplement: 1 [file NIHMS1534424-supplement-1.tif]

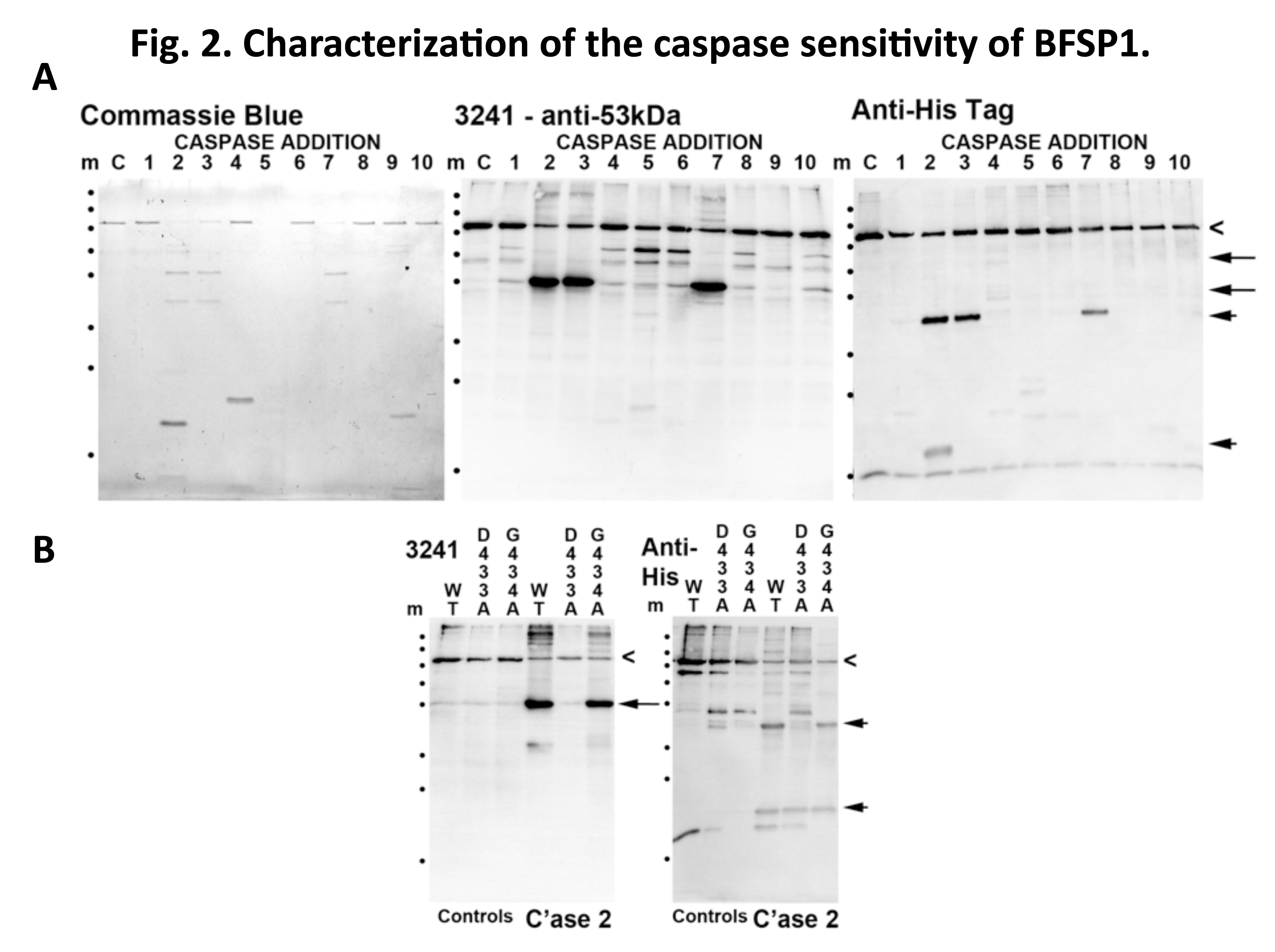

Supplement: 2 [file NIHMS1534424-supplement-2.tif]

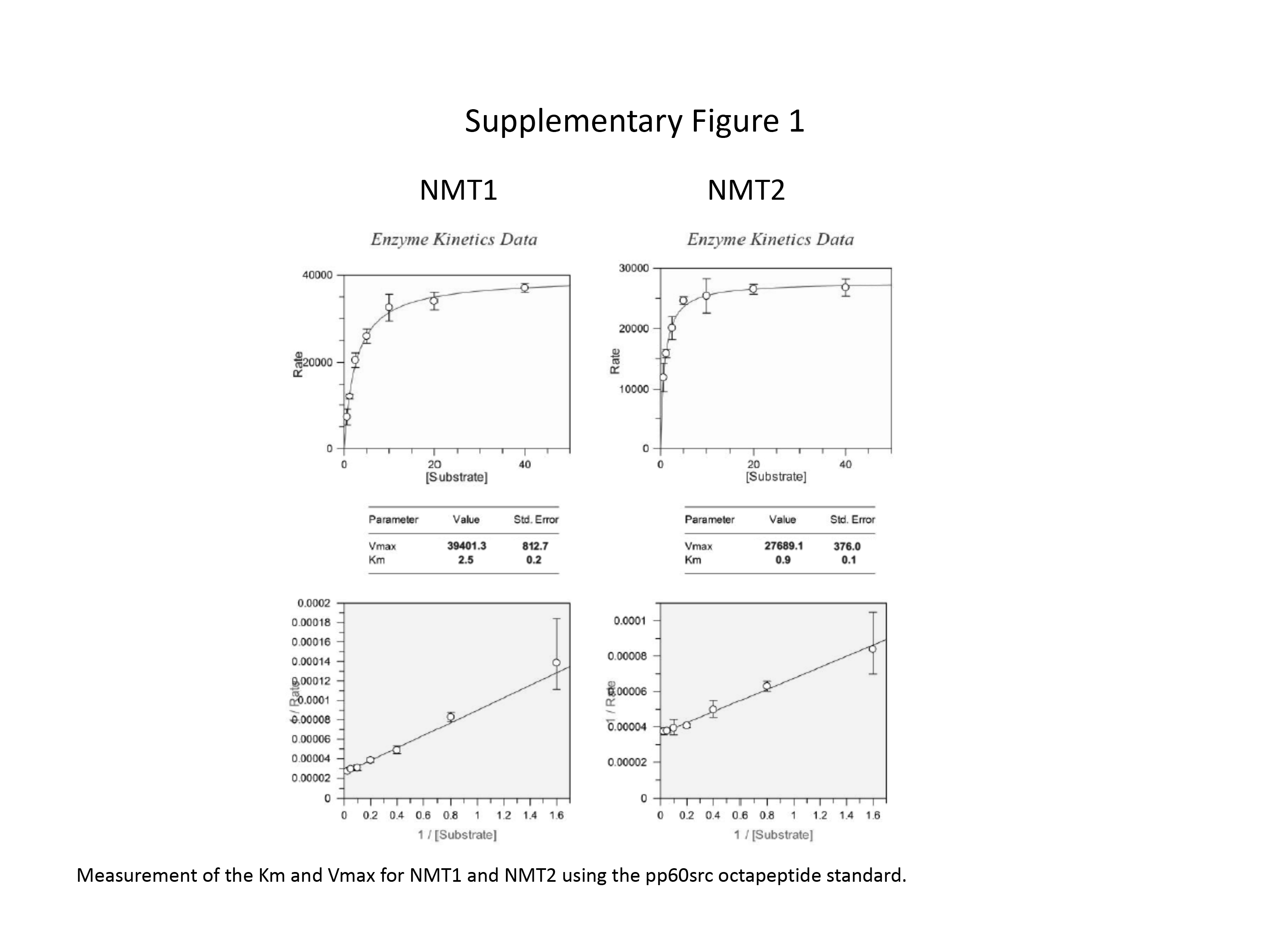

Supplement: 3 [file NIHMS1534424-supplement-3.tif]

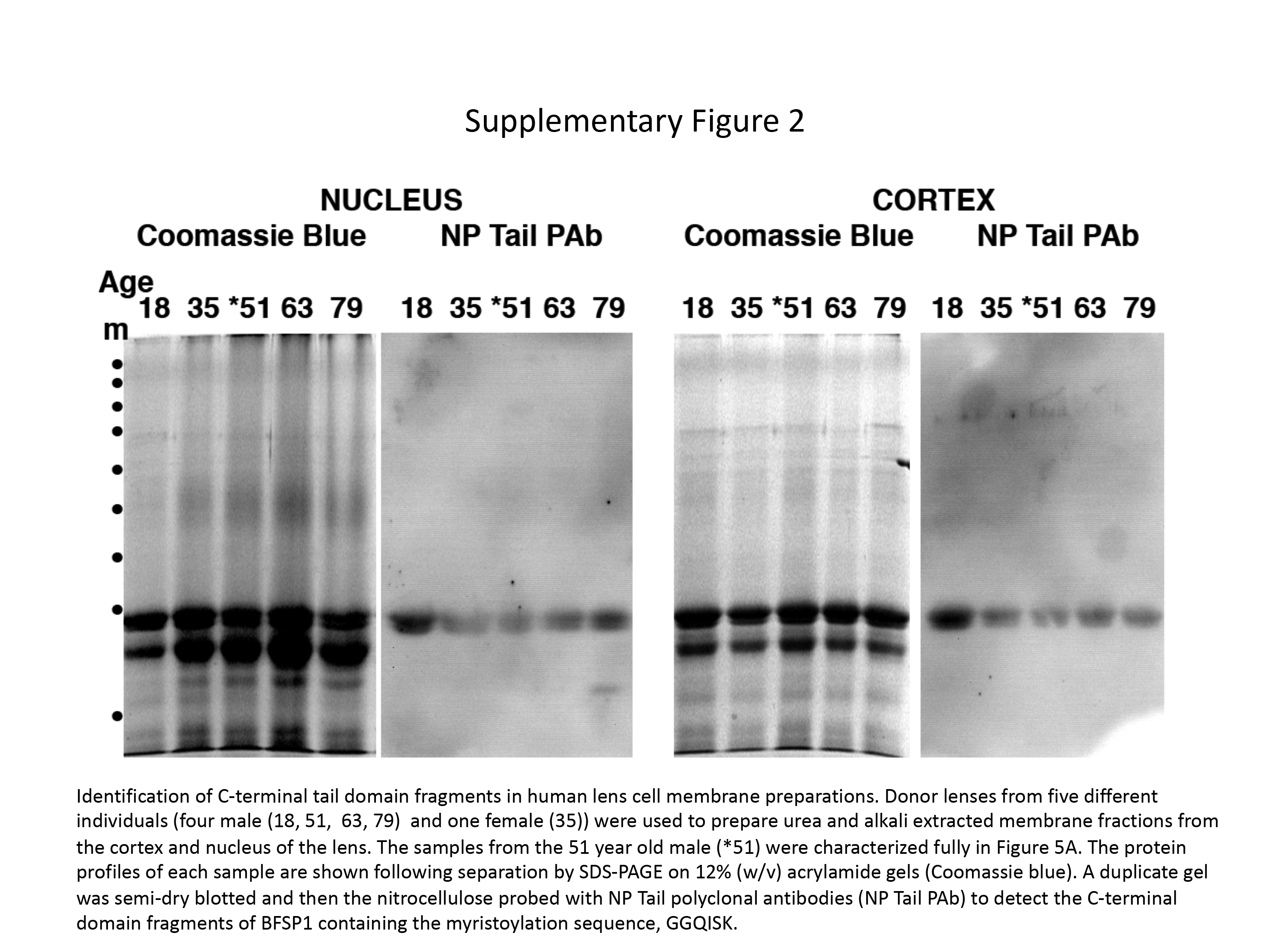

Supplement: 4 [file NIHMS1534424-supplement-4.tif]
